# What Bias Management Can Learn From Change Management? Utilizing Change Framework to Review and Explore Bias Strategies

**DOI:** 10.3389/fpsyg.2021.644145

**Published:** 2021-12-15

**Authors:** Mai Nguyen-Phuong-Mai

**Affiliations:** Amsterdam University of Applied Sciences, Amsterdam, Netherlands

**Keywords:** change management, behavioral change, cultural change, stereotype, implicit bias, organizational neuroscience, dynamic paradigm, bias intervention

## Abstract

This paper conducted a preliminary study of reviewing and exploring bias strategies using a framework of a different discipline: change management. The hypothesis here is: If the major problem of implicit bias strategies is that they do not translate into actual changes in behaviors, then it could be helpful to learn from studies that have contributed to successful change interventions such as reward management, social neuroscience, health behavioral change, and cognitive behavioral therapy. The result of this integrated approach is: (1) current bias strategies can be improved and new ones can be developed with insight from adjunct study fields in change management; (2) it could be more sustainable to invest in a holistic and proactive bias strategy approach that targets the social environment, eliminating the very condition under which biases arise; and (3) while implicit biases are automatic, future studies should invest more on strategies that empower people as “change agents” who can act proactively to regulate the very environment that gives rise to their biased thoughts and behaviors.

## Introduction

In daily life, we constantly make judgments and deal with uncertainty without knowing their outcomes. Three central mechanisms to make decision are: logic, probability, and heuristics. Logic focuses on truth, probability on statistically best “bets,” and heuristics on a good-enough solution (Gigerenzer, 2008). Humans often violate the rules of logic and probability, and resort to heuristic decision making. Such a mental shortcut provides a rapid and effective access to knowledge in order to deal with a social category. There are four main reasons for heuristics to exist: (1) in terms of “cognitive psychology” because human processing capacity is limited (Kahneman, 2003); (2) in terms of “neurobiology” because of the inherent design characteristics of our brain (Korteling et al., 2018); (3) in terms of “ecology” because these mental shortcuts are effective in their own right (Gigerenzer, 2000); and (4) in terms of “evolution” because heuristics are adaptive responses that promote humans’ fitness (Gigerenzer, 2008).

While heuristics have benefits, in the modern society and the context of interpersonal and intergroup relations, their effects can be deleterious. Implicit biases against stigmatized others are forms of heuristics that have a profound consequence on judgments and behaviors (e.g., Derks et al., 2008; Kubota et al., 2013). Their destructive power comes from three main features: (1) the computation of fitting people in the box is fast and automatic, to the extent that 100ms is enough to draw a judgment about a stranger’s face (Willis and Todorov, 2006); (2) they influence sensory predictions, altering “reality” to fit people’s expectation, dictating how others “should appear” (Brooks et al., 2018); and (3) they may involve selfless behaviors, for example, White participants were willing to discriminate against Black participants, even at a cost to their own financial gain (Kubota et al., 2013).

Twenty years since the Implicit Association Test (IAT), a plethora of studies have focused on strategies to reduce this biased tendency. However, while implicit biases can be reduced, the effectiveness of those strategies and reduction on behavior is inconsistent. To offer a clearer picture of this inconsistence, a systematic review of 494 studies by Forscher et al. (2019) concluded that there is little evidence that a reduction in implicit biases will translate into changes in behaviors. This is problematic, because while a bias is a mental inclination, discrimination is biases in action. In other words, one’s behavior causes discrimination. It is worrisome knowing that strategies that could reduce implicit biases in the mind may not necessarily lead to reduced discriminatory behaviors in real life. Also, as pointed out in the introduction of this research volume, the increasing popularity of implicit measures magnifies the inconsistence and misunderstanding on how to change implicit biases and their behavioral consequence. Lacking evidence of direct effect on behaviors thus makes strategies aiming at reducing implicit social cognition face more challenges. To address this thorny question, reviews have suggested improvement in methodologies, pools of participants, categories of biases, implicit vs. explicit measures, and especially caution in using the IAT as a behavioral predictor (e.g. Amodio, 2008; Greenwald et al., 2009; Cameron et al., 2012; Carlsson and Agerström, 2016; Forscher et al., 2019). But most importantly, FitzGerald et al. (2019) suggested a paradigm shift, focusing on how to reduce the negative impact of implicit biases in behaviors rather than just reducing implicit biases themselves.

This paper took on the suggestion of FitzGerald et al. (2019) to explore bias strategies that aim at a behavioral change. The paper’s method was inspired by an interdisciplinary review of Cikara and Van Bavel (2014). Such a holistic approach allows us to involve other disciplines and broaden the possibilities. The proposal here is, if the major problem of implicit bias strategies is that they do not translate into actual changes in behaviors, then it could be helpful to learn from change management. First of all, biases and habits are similar in the sense that they both function like an ingrained pattern of thought and behaviors (Cox et al., 2012; Devine et al., 2012). Second of all, change management has a strong focus on explicit changes at both individual and organizational level (Buckworth et al., 2007; Burke, 2017), which is not too far from a goal of bias management in term of aiming for actual changes in behaviors. Third of all, this discipline strives for systematic changes rather than a quick fix, which also addresses a major limitation of implicit bias interventions where 97% of the studies used procedures that took one single experimental session to complete (Forscher et al., 2019). The shared idea between change management and studies of implicit bias strategies is that “people can change,” and if change management has successful strategies, then bias management could benefit from this wide array of concepts and methods that change management and adjunct disciplines have to offer.

Thus, this paper uses a theoretical framework of change management to explore suggestions from interdisciplinary studies that could be potential to improve and develop bias strategies that result in a change in behavior. This allows a multifaceted exploration of findings from many fields of study that have contributed to successful change interventions such as reward management (Milkovich and Newman, 2002; Buckworth et al., 2007; Lewis et al., 2016), social neuroscience (Amodio, 2008), health behavioral change (Prochaska and Velicer, 1997; Kanter et al., 2010; Forbes, 2020), and cognitive behavioral therapy (Cox et al., 2012). Such an interdisciplinary approach can help to reconcile seemingly discrepant findings and frameworks, providing new insight, offering hybrid solutions, and highlighting areas where future research may need to focus on. It’s also argued that this integration could help us to move beyond mere describing and reporting the effects of a specific bias (e.g., ingroup bias), a specific population (e.g., women vs. men), or a specific method (e.g., neuroscience and social science).

In the following sessions, the paper briefly introduces some basic taxonomies of change management frameworks and the selection of one model for this paper. The purpose is not to choose the best change model. Instead, the purpose is to find one that could act as a “platform” or “sorting categories” that enable the incorporation of findings across diverse disciplines, helping us to yield new insight. The paper then continues with its review, followed by a discussion and suggestions for future research.

### A Change Management Framework

Change management is defined as the process of continually renewing an organization’s direction, structure, and capabilities to serve the ever-changing needs of external and internal forces (Moran and Brightman, 2001). It emerged as a discipline after the Second World War thanks to the rapid economic development which required organizations to effectively cope with changes in sizes and complexity (Burke, 2017). The bewildering array of change literature evolves from three different precursors. Firstly, Taylor (1911) viewed the organization as a machine, and change as scientific management. This precursor paved ways to modern processes such as total quality management (Corbacioglu, 2016). Secondly, the human relations movement arose as a counterpoint of the first approach (Collins, 2005). Originated in the Hawthorne effect, the founder of this movement – Mayo (1933) – viewed change in the context of human’s wellbeing and social aspects rather than individuals as merely components in organizational system that focuses mainly on higher output. While this movement has been contested (see for example, Purser, 2000), it paved way to theories and practices that promote change based on involvement and interpersonal skills (Collins, 2005; DuBrin, 2007). And thirdly, Kübler-Ross (1969) created a model of grief management based on how humans emotionally responded to the trauma of change. This precursor was adopted in other disciplines and laid foundation for many organizational studies because of its focus on how to help employees cope with loss and consequences during a change process (Castillo et al., 2018).

From these early fields of thought in change management, many rich lines of change literatures have emerged, covering a wide range of change aspects such as anticipation, diagnosing, planning, implementation, communication, motivation, measurement, evaluation, sustaining, resistance and politics of change (see for example Hayes, 2018; Deszca et al., 2019). However, because the foundation precursors of change management have placed a strong focus on processes, the central stage in change literature tends to be dominated by different models, frameworks and roadmaps of change. These models, frameworks and roadmaps can be divided into two broad categories. Firstly, there are organizational frameworks that provide a structural approach for institutional change, among them the most prominent are those proposed by Lewin (1947) and Kotter (1996), the “Prosci ADKAR” by Hiatt (2006), the “Transition Model” by Bridges (1995), and the “McKinsey 7-S Change Model” by Waterman et al. (1980). These frameworks emphasize different aspects and their application differs in different circumstances (Galli, 2019). Secondly, there are frameworks for individuals that can subsequently support organizational change management or incorporated in the organizational change process. They include the “Change Curve” by Kübler-Ross (1969), the “Seven Habits” by Covey (1989), the “Nudge Theory” by Thaler and Sunstein (2009), the “Switch Method” by Heath and Heath (2008), the “Habit Loop” by Duhigg (2012), and the “STREAP-Be” model by Nguyen-Phuong-Mai (2019).

The purpose of this paper is to explore insights that change frameworks can offer bias solutions in terms of changing biased behavior. Taking this into account, three selection criteria were established: (1) the selected change framework does not strictly adhere to the scientific management perspective rooted in the theory of Taylor (1911), which may require sequences of steps. Such a rigid structure may also lack a focus on humanistic aspects such as emotions, and hence, could compromise a dynamic application of findings from different disciplines; (2) the selected framework is not too generic, providing categories that could be too liberally interpreted, for example the model Lewin’s (1947) of three steps “unfreezing,” “changing” and “refreezing.” However, it shouldn’t be too complex, creating narrow and specific management categories which could render findings from other disciplines irrelevant; and (3) the selected framework is applicable for both individual and collective change management. This criteria is important, because many change frameworks have been designed only for organizational transformation, while for this paper, the aim is to explore bias strategies that are relevant for both collective and individual level.

To align with these three established criteria, the STREAP-Be framework (Nguyen-Phuong-Mai, 2019) was selected. The acronym stands for critical elements that could be taken into account in order to change successfully: “S” for dealing with *safety* issues, “T” for dealing with *triggers* that could (un)consciously activate a change or a habit, “R” for optimizing different kinds of *rewards*, “E” for strategies that deal with *emotions* as drivers of change, “A” for *aligning* with goals, meanings and values that motivate people to change, “P” for a focus on *people* that could be change agents, and “Be” for a focus on specific *behaviors* that lead to change. Each of these components is clear and straightforward in their indication. Table 1 provides a summary of this model.

**TABLE 1:** STREAP-Be model of 7 focuses in change management (Nguyen-Phuong-Mai, 2019).

*STREAP-Be: 7 focuses of change management*	
*S for “Safety”*	Rationale: Fear can hinder sustainable change.
	Strategy: Tackle safety/fear issues that make people (1) hold onto old habits and (2) avoid new ones.
*T for “Trigger”*	Rationale: Triggers activate habits (un)consciously.
	Strategy: (1) Eliminate or re-purpose triggers that activate old habits and (2) create new triggers that guide the formation of new ones.
*R for “Reward”*	Rationale: Rewards provide positive reinforcement to change.
	Strategy: (1) Minimize rewards of old habits and (2) create new rewards that motivate and reinforce new ones.
*E for “Emotion”*	Rationale: Emotion is the driver of motivation and the “fuel” for change.
	Strategy: (1) Manage emotions that make people hold onto old habits and (2) induce emotions that are strong enough to drive new ones.
*A for “Alignment”*	Rationale: People are hard-wired to align with and change for ingroups and values. But these ingroup boundaries and value constructs are soft-wired.
	Strategy:(1) Identify ingroups and values that make people hold onto old habits and (2) create new ingroups based on similarities, promote values that people can align with and unite to change.
*P for “People”*	Rationale: Change agents and positive interpersonal contacts drive change.
	Strategy: Work with influential people(1) who perpetuate old habits and (2) those who can lead and role-model the change.
*Be for “Behavior”*	Rationale: Actions beget motivation. The body leads, the mind will follow.
	Strategy: Identify desirable behaviors to track and reward them. Break down to mini goals, small wins, progress not perfection. “Fake” it until you *become* it.

Again, it is emphasized that the purpose is not to select the best change model. Instead, STREAP-Be was chosen because its structure is dynamic, providing categories and components that could open up a rich and dynamic platform that allows exploration and incorporation of various interdisciplinary findings. For example, the “S” for safety could be a platform to discuss many change management studies in psychology, neuroscience, management and organizational studies. In the following sessions, the STREAP-Be will be used as a *sorting framework* to (1) integrate studies from different fields and (2) hypothesize their potential on implicit bias strategies that could lead to changes in behaviors at both individual level and collective level.

## Utilizing a Change Framework as a Platform to Review and Explore Bias Strategies

### The “S” of STREAP-Be: Safety Strategies

Psychological safety and fear issues are major aspects in the literature of change management (Kiefer, 2002). Organizations and individuals need to identify and deal with fear, anxiety, or threat issues that may hinder them to change. From evolutionary point of view, fear increases vigilance toward aversive stimuli and enhances the ability to detect and avoid danger (see Baumeister et al. (2001) for a review, but also consider Corns (2018) for a counterargument). For survival purposes, the brain prioritizes fear and may register it before consciousness (Burra et al., 2013). Notwithstanding its evolutionary benefits, fear also impairs proactive and enhances reactive control (Yang et al., 2018). Taking into account this two-side effect of fear, this session discusses potential safety/fear strategies in tackling implicit biases with an aim for a behavioral change.

#### Emphasize the Ability to Change

Implicit bias strategies often raise people’s awareness of biases’ automatic nature. This may create some psychological safety as people realize that such a tendency can happen unconsciously without harmful intention. However, the downside of awareness is that people may learn to normalize implicit biases, having less encourage to change, making them a conscious norm, even abusing it, and in effect, encouraging the expression of biases (Duguid and Thomas-Hunt, 2015). The activation of a negative stereotype increases stereotype-conducive behaviors (Campbell and Mohr, 2011).

To address this problem, studies on change management can offer some insight. On the one hand, because fear is rooted in a survival instinct, fear appeal does lead to a behavioral change. On the other hand, people fear the uncertainty and potential failure as a result of change (Kiefer, 2002) and in general, threatening messages are not effective (see Ruiter et al., 2014 for a review). To explain this conflict, a meta-analysis of Peters et al. (2013) pointed out that effect on behavior is more likely to occur when there is sufficient perceived “self-efficacy.” It refers to one’s ability to negate the harm and enact a response. For example, inducing fear of HIV infection is not effective, but the presence of counseling that enables people to increase their knowledge and practice of condom use is effective (Earl and Albarracín, 2007).

Applying this in the context of bias strategies, it’s argued that we should highlight both (1) the destructive consequences of implicit biases (i.e., fear) and (2) the capacity to override them and to change biased behaviors (i.e., self-efficacy). This resonates with the framework that Sukhera and Watling (2018) proposed, which emphasizes (1) “the influence of biases on self and others” and at the same time, (2) “conscious effort to overcome bias.” Interventions conducted by Carnes et al. (2015) confirmed this. It’s also supported by Carr et al. (2012), namely, being taught that prejudices were “malleable” lead to more positive interracial interaction compared to being taught that they were “fixed.”

To conclude, insight from change management suggests that a potential bias strategy that could lead to a behavioral change should emphasize (1) the harm that implicit biases do to the people themselves; (2) their own ability to negate the harm; and (3) the available support to enact an effective response, both at the individual and institutional level (e.g., counseling and instrument that support the change process).

#### Create a Safe Environment

Fear has been discussed intensively in implicit bias literature, however, mostly with a focus on “stereotype threat” behaviors. In essence, those who are reminded of their group’s supposed inferiority have to perform under three neural disruptions that tax the brain’s limited executive control strength: (1) A physiological stress response that impairs the prefrontal cortex; (2) a performance monitoring response that may block automatically effective routines; and (3) an emotional regulation response to suppress negative thoughts (for a review see Derks et al., 2008). This in turn, could contribute to poorer performance. This fear is subconscious, which explains why when being confronted with negative stereotypes, threatened women maligned their own ingroup in the neural report, but favored this very ingroup in their self-report (Derks et al., 2008).

To a lesser extent, fear is also discussed in terms of intergroup anxiety. It may arise in White people who feel anxious of being labeled as racist or untrustworthy (Shelton et al., 2010), leading to stronger pro-White score in the IAT (Frantz et al., 2004). Similarly, the more anxious police officers were about being seen as racist, the more likely they were to have used force against Black suspects (Goff et al., 2012), and thus, fear could be a justification for lethal force.

Fear of losing autonomy also exerts impact. For example, reading statements such as “We *should* all refrain from negative stereotyping” (versus “You are *free* to choose to value non-prejudice”) made people become more prejudiced than they had before (Legault et al., 2011). This may also explain why popular interventions such as diversity and cross-cultural training which often incorporates implicit bias awareness may pose anxiety problems, especially among the managers who risk feeling they are the source of the problems (Dobbin et al., 2007, 2015). In a (sub)conscious way, fearful participants may psychologically challenge the whole system, perpetuate the *status quo*, or sabotage for revenge (Dobbin and Kalev, 2016).

Bias strategies have addressed these fear issues. Firstly, creating an identity-safe environment can positively influence performance (e.g., Davies et al., 2005), promote a two-way dialog (Sharma, 2017) especially when implicit biases are used as a point of discussion (Sukhera and Watling, 2018). Secondly, the source of anxiety must be identified. For marginalized groups, it comes from stereotype threat (Derks et al., 2008) or tokenism (Cundiff et al., 2018), but for privileged groups, it is unintentional expressions of stereotypes (Shelton et al., 2010; Carr et al., 2012).

However, it’s likely that there could be more safety/fear issues involved and there should be strategies dealing with them. To investigate this hypothesis, the SCARF model by Rock (2008) is a potential candidate from the field of neuroleadership that may offer some interdisciplinary insight. SCARF refers to five primary safety conditions that should be assured for people to change without being held back by fear. They are (1) *status:* position in a hierarchy; (2) *certainty:* ability to predict future; (3) *autonomy:* sense of control; (4) *relatedness:* sense of attachment with other; and (5) *fairness:* perception of fair exchange. This model could act as a holistic framework to understand and address the psychological safety and fear issues that people may have during bias interventions. For example, in the diversity training as noted earlier, the safety issue could be (1) “status,” because privileged groups felt they were put in the spotlight for accusation; and (2) “autonomy,” because marginalized groups could feel that bias awareness is raised but there is not much they can do to mitigate the situation other than relying on the goodwill of others. Based on SCARF, interventions could be systematically redesigned to check, address, and provide solutions in all five safety issues, creating a more thoroughly change-supportive environment for every party involved.

Taken together, while fear can lead to behavioral change, it’s more likely to be effective when combined with an emphasis on self-efficacy. This insight from change management suggests that we should highlight the capacity to change as well as provide supportive system for that change to happen. Further, future research could use the SCARF model as a holistic framework to evaluate and (re)design bias interventions that ensure psychological safety for a behavioral change to happen, but at the same time, eliminate the possibility of normalizing implicit biases.

### The “T” of STREAP-Be: Trigger Strategies

For implicit biases, triggers are cues that automatically activate a mental association of a biased habitual response. For example, violent rap music triggered the activation of the amygdala and the dorsolateral prefrontal cortex (DLPFC) in White subjects (Forbes et al., 2012). In the literature for organizational change management, triggers are explicit drivers of change such as competitors, technology, efficiency, or policies (Dawson, 2002). At the individual, change literature refers triggers to cues that activate a habit (Duhigg, 2012). Combining the meaning of triggers in these disciplines, they could be understood as cues that (1) drive changes or (2) activate a habitual response. Taking into account this two-side effect of triggers, this session explores potential strategies that tackle implicit biases with an aim for a behavioral change.

#### Prime Counterstereotypes

Priming people with counterstereotypical triggers has been one of the most consistent interventions to reduce implicit biases (see FitzGerald et al., 2019 for a review). However, as pointed out in the review of Forscher et al. (2019), there was little evidence that a change in implicit bias would lead to a change in behaviors. Despite this damning conclusion, the review’s forest plot indicates that across all procedures, the most potential one in changing behaviors is strengthening association directly. Future studies could pay more attention to this direction and explore effective methods with a clear objective of changing behaviors.

With regard to successful strategies in behavioral change (e.g., Kawakami et al., 2007; Gocłowska et al., 2013), neuroscience offers some significant insight. Amodio and Devine (2006) suggested that (1) implicit stereotyping and (2) implicit evaluation have different neural substrates of memory systems and predict different kinds of behaviors. The strategies for the former should focus on repeated counterstereotypical parings of semantic (i.e., words) triggers. By contrast, the latter is driven by affective processes which involve the automatic nervous system and behaviors associated with threat, and therefore, strategies should utilize the multiple memory system model, affectively laden images and counterstereotypical experiences rather than words (Amodio, 2008). This could be a valid indication. For example, asking people to actively generate counterstereotypes rather than being passively primed resulted in increased creativity (Gocłowska et al., 2013). Similarly, participants in Kawakami et al. (2007) made “approach” versus “avoidance” movements while being presented faces of White and Black people. Those who were trained to approach Black people showed less discomfort during actual interaction with Black people. In short, because evaluative biases reflect affective processes such as the amygdala, strategies that involve multiple memory system model could be advantageous.

However, while insight from neuroscience may give useful indication for future research, more studies should also focus on an issue rarely discussed while counterstereotypes are utilized, namely, the “backlash effect.” As Rudman and Fairchild (2004) pointed out, perceivers were more likely to sabotage atypical group members, and atypical people who feared sanctions reacted in ways that would maintain cultural stereotypes. More understanding of such effect may explain why a reduction in implicit biases does not necessarily lead to a change in biased behaviors.

#### Prime Positive Identities

Increased cognitive performance could be the behavioral result of using positive identities as triggers. For example, priming Asian women to think about gender decreased their math performance, but priming them to think about race increased it (Gibson et al., 2014). Similarly, priming biracial individuals with either their mother’ or father’s racial identity resulted in different behavioral interaction strategies based on salient identities (Gaither et al., 2013). This malleability of identities is supported by both behavioral (see Cameron et al., 2012 for a review) and neural studies (e.g., Chiao et al., 2010).

However, for some marginalized groups, it could be challenging to find an identity that is positively perceived. Further, it is unlikely that such priming is associated with long-lasting behavioral change (Forscher et al., 2019). This paper proposes that future studies should explore the possibility of a shift from “priming identities” to “cultivating identities.” The rationale of this shift can be found in a rich line of research in Identity Based Motivation theory. It posits that people’s self-concepts of who they want to be will motivate and trigger them to take action toward how that identity is socially perceived (Oyserman, 2015). Literature on identity (re)construction is also abundant, for example, identity can be cultivated through discourse (Bamberg et al., 2011), self-regulation (Nurra and Oyserman, 2018) or participation in different communities (Blåka and Filstad, 2007). Moving from priming identities to cultivating identities takes time and may demand systematic restructure and cooperation of different stakeholders. However, the immense advantage of triggers when they are internalized as identities should not be undermined.

#### Use Action-Demanding and Goal-Driven Triggers

In change management, triggers (e.g., new laws, market demand, or employees’ satisfaction) are often explicit, systematic, urgent, imposing, and most important of all, action-demanding. One reason for this is because many organizational change initiatives are rooted in a problem-driven perspective (Staudenmayer et al., 2002).

Some bias interventions have a similar approach. For example, reflective discussion (Sukhera et al., 2019) and brief bias trainings in general (Carnes et al., 2015) act as a provocative trigger to foster engagement. However, the effectiveness of such training is debatable as they don’t always lead to a behavioral change (Noon, 2018). More comprehensive interventions that took place over a longer period with specific goals and guided actions could be more effective (e.g., Devine et al., 2012) but because of the immense variety, it is challenging for comparison and setting up a goal standard (Silverstone et al., 2013). The literature on bias training is fragmented, covering many disciplines, and thus, lacking a cohesive unifying framework that could guide multi-level strategies for individuals, organizations, communities, and the society at large (Sukhera and Watling, 2018). Future research bears the responsibility to contribute further solutions to this problem.

Bias management could also learn from change management by increasing a sense of urgency for change. For example, many organizations have been successful in creating explicit action-driven triggers for “diversity” by connecting it to internal and external pressure. These include demands from governmental laws such as affirmative action and quota (Gabaldon et al., 2017), demands from stakeholders to make workforce reflect customer base (Siperstein et al., 2006), demands of knowledge management when entering a new market (Wrench, 2008), and demands to beat competitors in retention of multicultural talents (Ng and Burke, 2005). These demands are relevant, concrete, with clear consequences if there is “no action” or if organizations “fail to act effectively.” Since bias management and diversity/inclusion are closely connected, to the point that implicit bias training is often part of diversity policies, it’s argued that the rich and practice-oriented literature on diversity/inclusion has much to offer on designing paramount and action-oriented triggers that drive people toward behavioral change in bias management.

As a specific example for such connection, it could be made clear to employees that the negative implicit biases against those with autism may result in a failure of seeing their cognitive capacity as an asset. This is a fact that many software firms such as SAP and Microsoft have capitalized on. Employees with autism achieved 48–140% more work than their colleagues (Krzeminska and Hawse, 2020). Thus, in this example, work efficiency acts as an explicit trigger, and the “Autism at Work” program of SAP makes it more concrete with a clear goal of having 1% of the total workforce fall on the spectrum.

Taken together, triggers are cues that (1) activate a habitual response or (2) drive a change. While using counterstereotypes and priming positive identities can lead to behavioral change, interdisciplinary insight suggests that future research could further explore: (1) a focus on a multiple memory system model that combines affectively laden counterstereotypical experiences; (2) a shift from “priming” to “cultivating” identities, thus encouraging individuals to act in accordance with their new identities; and (3) the use of action-demanding/goal-driven triggers with clear behavioral goals.

### The “R” of STREAP-Be: Reward Strategies

In change management, reward plays a central role in shaping behaviors (e.g., Milkovich and Newman, 2002; Buckworth et al., 2007; Lewis et al., 2016; Cameron and Green, 2019). As a “*quid pro quo*,” rewards provide positive reinforcement contingent on engaging in desired behaviors. Beyond change management, reward learning is the foundation to understand and influence human actions in general, which is “away from punishment” and “toward pleasure” (Schultz, 2015). The brain computes the value of a reward and translates into behaviors that allow an organism to get better rewards and win the evolution. This session explores potential reward strategies that tackle implicit biases and aim for a behavioral change.

#### Optimize Implicit Reward

In studies of implicit bias strategies, rewards are often implicit, in the sense that they are not explicitly used to motivate participants, and participants may not be aware of them. Rewards are associated with the striatum’s activation in the brain (Knutson et al., 2001) when observing “outgroup misfortune” and “identifying the self with an ingroup” (see Molenberghs and Louis, 2018 for a review). Being different from others alarmed the reward prediction system and guided people to make behavioral adjustment (Klucharev et al., 2009). Because social conformity is rewarding, people learn to unconsciously adhere to a certain collective perceptions, even when they are biased. Reward and risk can also be intertwined. In the economic game, for each additional dollar sent to a Black partner, brain activity increased more than it did for a White partner (Stanley et al., 2012). Less predictable rewards are more valuable, and the act of showing trust to less trusted others can be both risky and rewarding.

Despite promising implication, the impact of implicit reward on behavioral change has yet become a focal point of research. Because rewards are powerful in shaping approach behaviors, implicit rewards could be argued to influence behaviors in a deep, unconscious, and long-lasting manner. Fundamentally, reward pathways have evolutionarily motivated people and reinforced survival behaviors in everything from seeking food to having biases toward or against others (Schultz, 2015). This is why artificial intelligence has copied this implicit reward-rational choice from humans as a single unifying formalism to interpret behaviors (Jeon et al., 2020). Thus, future studies with insight from the neurobiology of reward may help to explore (1) which bias strategies are rewarding, and if so, whether they would be more effective as behavioral reinforcements; and (2) how we can optimize the power of implicit rewards in ways that motivate people to approach stimuli and engage in desired behaviors.

#### Optimize Explicit Reward

Explicit reward has been discussed in connection with ingroup or racial bias strategies. For example, reward structure mediated competition versus cooperation (e.g., Bettencourt et al., 1992), while financial incentives could actually worsen racial biases (Underhill, 2019). However, research on rewards as explicit motivators for behavioral change is still limited. It’s argued that bias management could benefit from rich lines of research on reward management, which covers invaluable resources of practices such as salary increase, bonus system, perquisite, promotion, status, authority, responsibility, education, appreciation, work-life balance, social activities, or feedback. People are capable of doing great things when they are rewarded for making a difference (Armstrong and Murlis, 2007). It could be a missing opportunity not to look at the insight that reward management has to offer, knowing the objective of this domain is to motivate people to change.

As a specific example, we could consider how the Self-Determination Theory underlines the strength of motivation (Deci and Ryan, 2012) and one of its novel applications in gamification. Points, achievements, badges, medals, animated feedback, kudos, and likes are rewards that could enhance perceptions of autonomy, competence and relatedness – all of which promote long-term behavioral change (Lewis et al., 2016). It could be argued that game-based learning may become a potential bias strategy. Researcher may design bias strategies in forms of personal apps in mobile phones – which is promising because it can be incorporated with other successful strategies such as counterstereotypes and implicit bias test. This also allows researchers to evaluate long-term effect among users – a weakness that bias strategies still have to deal with (FitzGerald et al., 2019).

Taken together, reward has not played a central role in bias strategies aiming at changing behaviors. If we acknowledge the power of reward in reinforcement learning, then all forms of rewards should be thoroughly explored. Insight from neuroscience, change, reward learning and reward management can significantly contribute to future research in this direction.

### The “E” of STREAP-Be: Emotion Strategies

The Latin root of “emotion” is “movere” (to move). In change management, emotion is the base of motivation, the “fuel” for change (Cameron and Green, 2019). A foundation theory for change management – the change curve of grief by Kübler-Ross (1969) – is also built upon emotional management.

Emotions drive implicit biases. For example, anger and disgust can create implicit biases against unknown groups where none existed before, and increase implicit prejudices against known groups (Dasgupta et al., 2009). In turn, implicit biases influence how one perceives emotion, manipulating sensory predictions, dictating how others emotionally “should” appear rather than how they “actually” appear (Brooks et al., 2018). For these reasons, emotions have been used to reduce implicit biases (FitzGerald et al., 2019). This session takes into account the two-side effect of emotions and explores strategies that use emotion as drivers for a behavioral change.

#### Induce Empathy and Perspective-Taking

Negative biases against others could be rooted in a lack of empathy – the capacity to share others’ emotional perspectives (De Waal, 2008). Hence, using empathy could tackle biases with a behavioral change. For example, writing a perspective-taking narrative essay about a young Black male strengthened automatic and positive behavioral approach toward Blacks (Todd et al., 2011). Similarly, emphasizing empathetic mindset among teachers reduced half of the suspension rate and improved teacher-student relationship for at-risk students (Okonofua et al., 2016). Fostering empathy also changed the behaviors of health care professionals to mitigate biases (Schwartz et al., 2020).

However, if we incorporate insight from other studies in change management, then a critical issue emerges. While inducing empathy may work as a bias strategy, little research has connected it with a possible impact of “empathy fatigue” or “secondary traumatic stress” among front-line professionals such as teachers, health care providers and social workers (e.g., Figley, 2002; Russell and Brickell, 2015). Even if empathy fatigue is unlikely to occur during brief interventions, for this bias strategy to work, empathy should become a habit, and thus, its long-term effect should be measured.

Next, we should pay attention to studies that pointed out the stark contrast in both psychological and neurological levels between “empathy” and “compassion.” While empathy is to “feel what others feel,” compassion is to extend loving and kindness to all human beings without preferences (Singer and Klimecki, 2014). While empathy led to an increased activation in brain areas associated with pain and self-reported negative effect, compassion increased positive effect in a neural network usually related to reward, such as the medial orbitofrontal cortex and the striatum (Klimecki et al., 2013, 2014; Kang et al., 2018). More importantly, while empathy fatigue can lead to withdrawal, compassion involves not only understanding but also *acting out* with a motivation to change the situation and help [Singer and Klimecki, 2014; however, see Luberto et al. (2018) for a mixed review]. Compassion, hence, could be a win-win strategy in which it enhances both positive emotions and prosocial behaviors in response to adverse situations (Leiberg et al., 2011; Weng et al., 2013).

This significant insight from neuroscience is potential of advancing the field of bias strategies far forward, because it could hit several targets at the same time: (1) reducing biases; (2) enhancing one’s own well-being; and (3) putting positive emotions in change action. Some studies support the first hypothesis (Lueke and Gibson, 2015; Kang and Falk, 2020). Many support the second (see a review by Galante et al., 2014). And initial findings support the third. For example, compared to Christian primes, those primed with Buddhist concepts that trigger compassion such as “reincarnation” and “monk” were more likely to tolerate outgroups and engage in prosocial behaviors (Clobert et al., 2015). Future studies should capitalize on this insight. It would be a significant shift in bias strategies if compassion can actually lead to behavioral changes while avoiding the possible emotional burden of empathy.

#### Induce Target Emotion

Empathy absorbs others’ feelings, both positive and negative. Specific emotions such as “guilt” and “shame” have been discussed. Focusing on the former inhibited ongoing biased behaviors, while focusing on the latter reinforced current behaviors (Fourie et al., 2014). People felt guilty when knowing that their (bogus) neural responses were biased, and when given an opportunity, they were more likely to engage in prejudice-reducing behaviors (Amodio et al., 2007). The indicative power of “guilt,” “shame,” and “love” (in compassion mindfulness) as emotional motivators of behavioral change suggests that other candidates in the basic emotion frameworks of Ekman et al. (1983) and Rozin and Royzman (2001) such as “pride,” “anger,” “disgust,” “sadness,” “surprise,” or even “fear” deserve more attention in future research.

Next, bias strategies could benefit from significant insight in the established field of “emotion management.” Strategies could be improved with the understanding of how emotions play a significant role in change processes, and how they could be a *valuable resource* readily for development (Ashkanasy et al., 2002; Bianchi et al., 2016; Cameron and Green, 2019). For example, at the organizational and societal level, management and leadership could be strategic in leading emotional reactions and galvanize people into actions (Thiel et al., 2012). After all, implicit biases do not rise in a vacuum. And so, emotions are more than a product of a specific individual but a result of dynamic interactions with the social environment. Toward that direction, future research has much to learn from organizational and emotion studies and to capitalize on their insight to advance.

#### Reappraise Emotion

It was stated earlier than stereotype threats impair intellectual performance due to the need to regulate emotion which reduces working memory efficiency. To deal with such a challenge, there are two common strategies: suppression and reappraisal. Suppressing emotion (1) inhibits inner feelings, (2) decreases outward expression but (3) fails to decrease emotional experience, (4) impairs memory and (5) has the same negative effect on executive resources as stereotype threat (Gross, 2002; Johns et al., 2008). By contrast, reappraisal changes the way a situation is construed and so, (1) decreases emotional experience, (2) decreases behavioral expression, (3) has no impact on memory and (4) shows benefit in dealing with stereotype threat (Gross, 2002; Johns et al., 2008). In practice, participants were asked to reappraise not the situation, but the anxiety they felt as a result of the situation, or interpret the stimulus in a way that alters their emotional reaction.

What is noteworthy is that while the literature on stereotype threat is abundant (Derks et al., 2008), studies on emotion management for those who are subjected to stereotype threats are in a disproportionally small number. Future research can fill this gap, taking insight from the rich literature on emotion management (see Bianchi et al., 2016 for a review) and the small but increasing number of studies on how mindfulness can regulate stereotype threats (Weger et al., 2012; Jarunratanakul and Jinchang, 2018).

Taken together, emotions as bias strategies can be significant drivers of behavioral change. While there is evidence that empathy and emotions associated with morality are promising solutions, by incorporating insight from other studies in change management, it’s suggested that future research could take an interdisciplinary approach and look further into: (1) the potential benefit of compassion vs. empathy; (2) the impact of other emotions beyond what have been studied such as “guilt,” “shame” and “love”; (3) the power of emotions not as an individual state of mind but a “collective resource” for bias management at organizational and societal level; and (4) the development of emotion management tool kit for those subjected to stereotype threats.

### The “A” of STREAP-Be: (Goals) Alignment Strategies

In change management, goal alignment is a critical approach because conscious goals affect actions (see Locke and Latham, 2002 for a review). With the “fuel” of emotions, humans are motivated to achieved three main goals: “communion,” “meaning” and “agency” (see Talevich et al., 2017 for a review). This session explores how bias strategies can emerge from the way people align with these three overarching goals, and as a result of that alignment, are motivated to change their behaviors.

#### Use Recategorization

“Communion” as a fundamental goal (Talevich et al., 2017) is rooted in how people instinctively want to align with an ingroup, hence, they categorize people, which could result in intergroup bias (Brewer, 1979). However, while intergroup is “hard-wired,” ingroup boundary is “soft-wired.” It took less than 4 minutes of exposure to a new group in order to deflate the tendency to categorize others by race built in a life’s time (Kurzban et al., 2001). The “minimal condition” for ingroup bias is simply being a member of an arbitrary group formed by, for example, flip of a coin (Tajfel et al., 1971).

This notion of common ingroup identity explains the success of an intergroup bias strategy called “recategorization” (see a review by Gaertner and Dovidio, 2005). In essence, people align with an ingroup when they share an identity or develop a dual identity with others. Putting people in a minimal group, making them align with new group’s identity by competing with other groups can influence people’s empathy (e.g., Han, 2015) and interactions (e.g., White et al., 2014) toward their new group members.

The literature on recategorization is rich (Gaertner and Dovidio, 2005), and it continues to develop with new methods (van Hoorn, 2018), components (Dunham, 2013), models (Roth et al., 2018), and especially the effect in long term and large scale, for example, how the Rwandan government promoted a single recategorization strategy to unite the country after the genocide (Moss and Vollhardt, 2016). This converges with the strategy of “priming positive identities” and the suggestion to move toward “cultivating identities” mentioned earlier as triggers for a behavioral change. There are also novel interventions and mixed findings of studies using drug, such as the administration of oxytocin – a hormone associated with facilitating social affiliation and prosocial behaviors (see Nave et al., 2015 for a review). Another hormone with impact on intergroup bias is testosterone, often wrongly associated with aggression, but has contextual impact on trusted behaviors (Dreher et al., 2016). For example, under the effect of testosterone, people were *more* generous if their status relied on being fair and honest, but were more likely to punish others when received unfair offers (Eisenegger et al., 2011; Dreher et al., 2016). This is promising because it means testosterone could potentially facilitate affiliation when the social setting is right.

Taken together, “communion” in the sense of belong to an ingroup is an overarching goal that people naturally want to align with. It is both instinctive and fluid, which brings about both opportunities and challenges. The globalized contemporary societies add new elements to the mix, with frequent changes of group memberships across all aspects of life: jobs, organizations, cities, and virtual communities (Roth et al., 2018). This means increased complexity but also malleability of intergroup biases. Future research should focus on mechanisms that exploit the best of these new elements, while provide solutions that could help to predict and tackle issues that come with them (Gaertner and Dovidio, 2005).

#### Use Egalitarian Value

The second overarching goal in the review framework of Talevich et al. (2017) is “meaning.” This motive safeguards human’s virtues, ethics, and duties. It probably explains why for those who strongly hold egalitarian ideas, they are in a “state of conflict” (Allport, 1954) when it turns out that despite their explicit ethical values, implicit biases persist.

However, it is also upon this fundamental goal of meaning that people can align with their value-driven thoughts and actions to inhibit or suppress biases. A chronic motivation of egalitarian values could dominate even the strongest and fastest-acting conflicting responses (Moskowitz et al., 1999; Glaser and Knowles, 2008). Even temporarily priming people with tolerance, respect, cooperation, multiculturalism, and Buddhist concepts could reduce implicit biases (see FitzGerald et al., 2019 for a review). Because the brain can detect the conflict between egalitarian values and biases and exert control (Kubota et al., 2012), egalitarian values can be *external* motivation, helping people to implement cognitive and behavioral control efforts (Dunton and Fazio, 1997; Plant and Devine, 1998).

Despite promising findings, few studies have clearly demonstrated the impact of egalitarian values as external motivation on changing behaviors. However, people tend to act on their belief (Uhlmann and Cohen, 2007). And as mentioned earlier, morality can regulate biased behaviors (Amodio et al., 2007; Fourie et al., 2014). Taking into account that “meaning” is a fundamental motive of human beings, goal-alignment with egalitarian values could be a promising strategy for future studies. As Bargh (2006) argued, research should advance to the “second-generation” of priming for social behaviors. It should move beyond the perception-behavior link and focus more on how (non)conscious motivational goal pursuit can *dominate* the behavioral priming influence and lead to appropriate actions.

Taken together, change management literature suggests that goal alignment is powerful in guiding actions. Three overarching goals universally pursued by people are: communion, meaning and agency. Applying this framework to bias strategies, there seems to be rich evidence on utilizing “communion” (i.e., aligning with an ingroup). However, it also appears that “meaning” (i.e., virtues, ethics, and duties) and “agency” (i.e., skills and competence) seem to be areas that could benefit from further studies. Future research could focus more on this direction, with extra consideration on the impact of globalization and frequent change in social identities.

### The “P” of STREAP-Be: People Strategies

The Stereotype Content Model of Fiske et al. (2002) suggests that biases could be associated with an automatic (de)humanization of others along the high vs. low level of warmth and competence. For example, African Americans were perceived as low in both warmth and competence, thus being dehumanized (Fiske et al., 2002). This dehumanization process is associated with weaker activity of the medial PFC (Harris and Fiske, 2006) – a brain region linked to mentalizing and monitoring the integration of one’s action and actions of others (Amodio and Frith, 2006). Taking this into account, bias strategies should lead to re-humanization, so targets of stigma could be viewed as worthy of mentalizing and social engagement (Amodio, 2008). In the clinical context, mentalization-based treatment has been successful in creating behavioral change (see Malda-Castillo et al., 2019 for a review). The theory underlying the powerful impact of mentalization is the “Contact Hypothesis” (Allport, 1954). This session looks at strategies stemming from this theory and explores how they could benefit from other disciplines’ insight.

#### Optimize Positive Intergroup Contact

The contact theory enjoys a rich line of research showing the benefit of “positive intergroup contact” in improving humanness attributions and intergroup relations (see Dovidio et al., 2017 for a review). Neural studies backed this up. For example, as the result of life experiences, Asians brought up in Western countries and identified themselves as Asian showed comparable empathic neural responses to same-race and other-race models (Zuo and Han, 2013) – an effect that could happen in new immigrants within 5 years of arriving (Cao et al., 2015).

#### Individuate

“Individuation” as a strategy is rooted in the hypothesis that not quantity, but quality of contact than mere familiarity is a critical factor in reducing biases. For example, trying to perceive someone as a *unique person* influenced the amygdala’s activation and, potentially, regulated implicit biases (Wheeler and Fiske, 2005). This resonates strongly with emotion strategies in one of the previous sessions. Attempts to humanize others, taking a perspective approach, trying to individuate and empathize could be a powerful bias strategy with evidence in behavioral change (Todd et al., 2011; Okonofua et al., 2016; Schwartz et al., 2020).

#### Brain Stimulation

“Brain stimulation” targets the mPFC and could decrease implicit biased attitudes toward out-group members (Sellaro et al., 2015). On the one hand, replication is needed, and the effect on behavioral change has yet to be seen. On the other hand, the emergence of brain training techniques (e.g., Abend et al., 2019) means this direction could be promising.

In sum, contact hypothesis has been a foundation theory for a number of solutions. While the literature on contact theory and bias strategies continues to grow, one pressing question is how contact theory can become “fit for purpose” in globalized societies where group boundaries are more dynamic and actions for collective change become more critical (Dovidio et al., 2017). Future research needs to provide better understanding of how positive intergroup contact may create misleading perceptions of equality. In other words, how it may help to achieve better relationship but fail to influence societal changes (Everett and Onu, 2013). Because human contacts can both inhibit or promote actions for social change, we could not assume that positive intergroup attitudes would translate into positive intergroup behaviors and ideological changes at the collective level.

It is with this concern that change management may contribute to the future research on contact. A critical aspect of change management is a strategic focus on people as “change agents” (Ulrich and Brockbank, 2005). Their jobs are to develop a climate of transformation by overcoming resistance and rallying forces for positive change. They are pro-active, goal-aligning, action-oriented, and they are armed with skills, tools, and a purpose (Jabri, 2010; Lunenburg, 2010; Bartunek, 2014).

If we incorporate this notion of change agent into studies of contact, there could be benefit in the way people individuate, interact, empathize with others, and how this could lead to a wider societal change. Harking back to the motivation/goal framework of Talevich et al. (2017) in the previous session, the proposal here is, when motivated by “meaning” and “agency,” people may adopt a different mindset and create different impact. History has witnessed many individuals whose strong will to change the society has created landmarks in the landscape of intergroup relations. People also look up to role models and modify their thoughts and behaviors. So instead of creating a setting for positive contact to happen, and expecting that people and society will change, another approach is priming/training people to be change agents and to optimize that setting to promote change at both individual and societal level. For contact theory, change is the consequence. For change theory, change is the purpose. The combination of these two may create positive outcomes, and the “change agent mindset” could very well be a hypothesis that future studies in bias management may explore.

### The “Be” of STREAP-Be: Behavior Strategies

It’s argued that people unconsciously “know” they are biased (Neys et al., 2008, but see different view by Botvinick et al., 2001). But despite this warning, many “behave against their better judgment” (Denes-Raj and Epstein, 1994, p. 819) and fail to block this biased reaction. This means while implicit biases are automatic and strong, controlled processes do not demand a great deal of deliberation, and thus, it is practical and possible to focus on an ability to control biases with interventions designed to enhance the sensitivity of this system for example, with conscious deliberate actions (Amodio, 2008). This resonates with the impact of human “agency” proposed by the framework of Talevich et al. (2017) mentioned earlier. This session reviews bias strategies that optimize bias control with an aim for a change in behaviors, which will be grouped into two clusters: “deliberate control” and “action orientation.”

#### Deliberate Control

“Implementation intention” is a bias strategy that involves a mental rehearsal of an “if-then” planned response that connects a goal-directed behavior to a specific trigger. This was effective in a Shooter Task where participants made fewer mistakes of shooting Blacks without guns (Mendoza et al., 2010). It resonates with a strategy to reduce racial anxiety called “social scripts.” They are specific guidelines on appropriate behaviors, aiming to prepare people for intergroup contact (Trawalter et al., 2009). Another strategy was to ask people to “stop and think about their decision” for at least 10 seconds (Hughes et al., 2017). This tracked the ACC’s activity, often associated with conflict monitoring, and led to increased trust toward outgroup members in an economic game. Such findings in behavioral change indicate that people can override biases through deliberate effort. Further studies should further explore interventions that employ the “system 2” processing (Kahneman, 2003) which might result in behavioral change *without* having to change one’s implicit biases and affective experiences.

#### Action Orientation

Studies have suggested that once making a behavioral choice, even between equally attractive options, people are more likely to associate their attitudes and behaviors with that choice (Nakamura and Kawabata, 2013; Harmon-Jones et al., 2015). And because actions beget motivation, this could explain a bias strategy in the study of Estes and Felker (2012). Women did worse than men because they had given up halfway. When researchers ran the test again and asked *everyone* to attempt every puzzle, women did just as well as men. Confidence and performance drive each other. When people act, even if it’s involuntarily, the performance could increase. In turn, better performance can bolster confidence, stimulating even more practice to win back confidence.

The power of strategic actions enjoys decades of mental health research under the term “behavioral activation.” It posits that engagement in rewarding activities is a powerful mechanism for change (see Forbes, 2020 for a review in clinical context). Strategies associated with this theory have concrete structures such as activity monitoring, assessment of goals and values, activity planning, skills training contingency management, and many other processes aiming at (non)verbal behaviors (see a review by Kanter et al., 2010). This rich line of studies on behavioral activation is an invaluable resource for bias strategies that aim for behavioral change. As Cox et al. (2012) suggested, tackling prejudices and fighting depression have the same enemy, and thus bias strategies could very well benefit from evidence-based treatments used in mental health research. The interdisciplinary approach that Cox and colleagues employed resulted in several deliberate control and action-oriented bias interventions such as “distancing and mental experimentation,” “thought record,” and “embodiment.” Their call for future studies to widen the lens and adopt an integrated perspective in order to pack a greater punch against personal and societal ills resonates strongly with the spirit of this paper.

## Further Discussion

This paper conducted a preliminary review and exploration by placing bias strategies within a framework from change management. By doing so, it wishes to incorporate new insight from studies that have contributed to successful change interventions such as reward management, social neuroscience, health behavioral change, and cognitive behavioral therapy. This session discusses several issues emerged from this interdisciplinary approach, its limitation, and future direction.

### Potential Strategies as Result of Interdisciplinary Approach

First of all, by utilizing a change framework as a platform for literature incorporation, previous sessions have demonstrated that many current bias strategies could fit well into the seven components of “safety,” “trigger,” “reward,” “emotion,” “(goal)-alignment,” “people” and “behavior.” Such a juxtaposition and calibration also revealed different levels of attention that research has invested into the understanding of these components. For example, trigger-strategies (e.g., counterstereotypes) and people-strategies (e.g., contact hypothesis) benefit from rich lines of studies. By contrast, other strategies regarding “safety,” “reward,” “emotion,” “goal-alignment” and “behavior” do not equally have the same attention, despite them being critical elements in change management and other adjunct disciplines.

Second of all, by incorporating different disciplines, new terminologies and concepts also ignite new insight and potential approaches. Regarding “safety,” (1) the SCARF model could be a holistic framework to (re)design inclusive psychological safety for all parties involved; and (2) abundant research in threat appeal strongly suggests that an emphasis on self-efficacy should be coupled with awareness of the harm caused by bias consequences and available support to enact behavioral change. Regarding “trigger,” (1) identities are powerful in changing behaviors and thus, a shift from “priming” to “cultivating” identities could influence biases more significantly, and (2) triggers for a change in biased behavioral could be goal-driven, action-demanding in the same way that triggers in change management usually mean. Regarding “reward,” (1) neuroscience holds an unmatched potential to understand and optimize implicit reward; and (2) rich lines of research in reward management indicates more focus on how explicit rewards can lead to behavioral change. Regarding “emotion,” (1) compassion seems to not only reduce biases but also promote pro-social behaviors – a limitation that empathy has to deal with, and (2) bias strategies could learn from how change management utilizes the power of emotions not as an individual state of mind but a “collective resource.” Regarding goal “alignment,” bias strategies could benefit from the overarching motives of people to intrinsically pursuit egalitarian values in virtues. Regarding “people,” abundant research in change agent suggests that when motivated by a sense of agency, people may adopt a different mindset and create different impact, thus transforming themselves and the society at large. Finally, regarding “behavior,” (1) the same sense of agency encourages more focus on bias strategies that capitalize on people’s deliberate actions, and (2) abundant research in “behavioral activation” could enrich bias strategies with elements of a successful intervention for behavioral change.

Taken together, it seems potential that strategies for a complex social phenomena such as social biases could benefit from an interdisciplinary approach. However, the employment of STREAP-Be in this paper is just one single example of how a strategic juxtapose and calibration of theories can reveal interesting angles that may deserve further investigation. Naturally, this choice of framework has its shortcomings. Firstly, this preliminary phase risks a lack of rigorous connection between different disciplines, and hence, in some cases, could only draw potential hypotheses instead of a clear implication. Secondly, this preliminary phase does not clearly indicate the differences between potential strategies for change at the individual and collective level. Thirdly, this top-down approach of reviewing studies sets a boundary and limits the power of exploration. This means a number of bias strategies that recently emerge or position themselves in the nexus of different disciplines could not be categorized. For example, this includes bias interventions that directly influence organizational structures by strategically changing diversity-related dimensions (Feng et al., 2020), or how artificial intelligence can tackle behavioral consequences of biases (Lin et al., 2020). Further, change management as a discipline has its own challenges as high rate of failure, contradictory approaches and a lack of empirical evidence (By, 2005). This means the framework used in this paper could be subjected to the same limitations of the discipline.

For those reasons, the paper will fulfill its purposes if similar integration could be carried out with other relevant change management frameworks and theories. Ideally, this should be done in a more systematic approach, looking for (in)consistent findings across different fields of study, gaining “consilience” at multiple level of analysis (Wilson, 1998). It is the power of interdisciplinary approach that will allow us to seek new solutions that could only be possible by a synergy of methods that traverse multiple levels in different study fields. After all, it is also social neuroscience and its interdisciplinary approach that gained us great insight in dealing with biases.

### Toward a Holistic and Preventive Approach

The previous session suggested that an interdisciplinary approach can enrich implicit bias strategies. Employing such an approach also helped Kahn (2017) point out a potential issue in how anthropologists and neuroscientists differ significantly on bias strategies. The former see negative biases as relational concepts grounded in the historical context (Mullings, 2005). The latter took history out of the focus and tend to biologize them with an emphasis on their automaton, making them a natural feature of life, a “timeless attribute in the human brain” (Kahn, 2017, p.271).

This discrepancy shapes responses to the problem. Neuroscientists tend to see implicit biases as a physiologically manifest process. Hence, they can be technically investigated and fixed with interventions, some of them were discussed in this paper, such as priming, emotion inducing, brain stimulations or drugs. For example, the impact of a drug called propranolol on the amygdala and implicit bias (Terbeck et al., 2012) was seen as “instrumentally good,” a “moral bioenhancement” and could be applied widely (Douglas, 2013; DeGrazia, 2014). In the same way that painkiller reduces the pain without solving the root cause, propranolol can mitigate racial aversion without correcting the beliefs. In short, implicit biases could be naturalized as a technical problem that might be addressed through a private means (Kahn, 2017).

Critics argued that adopting such a biological/physiological approach to solve ethical issues may undermine biases as “socially and historically situated manifestation of power relations” (Kahn, 2017, p.265). If “a pill a day keeps the prejudice away,” we may risk sidelining the root cause of the problem in power structure, cultural relation, and systemic discrimination. This could lead to the underestimation of collective solutions such as sustained social and political actions to achieve social justice. Just like painkiller does not solve the root cause of pain, successful but temporary interventions do not fix the root cause of biases. Once participants leave the experiments, they are back to a culture full of trigger stimuli that reactivate and maintain the very biases that were previously reduced in the lab.

Many interventions reviewed in this paper still linger on such a “technical fix” and “private means” approach. Taking this argument into account, there are reasons to reevaluate bias strategies in connection with collective actions and a wider social context rather than individual change. Holistic strategies should (1) incorporate multiple strategies and (2) aim at institutional changes such as collecting data to monitor equity, utilizing technology to aid objectivity, creating bias-free artificial intelligence, or blind evaluation. The connection between bias management and human resources, organizational studies and public policies should be much stronger in order to achieve significant and long lasting effect in the real world context.

To conclude, a sustainable solution may need the elimination of the very condition under which biases arise. As Huebner (2016) and Dasgupta (2013) argued, an egalitarian culture creates unfavorable environments for adverse biases to form in the first place. In other words, the optimal solution is a *preventive solution*. This resonates with the “goal-alignment” strategies with egalitarian values mentioned earlier. Short-term success has come from priming people with tolerance, respect, cooperation, multiculturalism, and Buddhist concepts (FitzGerald et al., 2019). To break free from the limitations of a “technical fix” and aim for a significant change, we can embrace the preventive approach and actively “engage in collective prefigurative practices designed to create a world where our reflexives reactions are already calibrated against our reflectively held goals and values” (Huebner, 2016). Along the line of the “change agent” mindset, we want to have the agency in ways that we are not dominated by reactions that we can’t reflectively avow. To quote Huebner (2016) again, people have to live as if an egalitarian world exists before one can actually does.

### Culture as Dynamic and the Role of People as Change Agents

To continue with this argument, taking one step further, such a holistic preventive approach can also galvanize our critical understanding beyond the boundaries of bias management and into the realm of collective norms and societal changes. Here is the line of reasoning: If cultivating a culture of egalitarian values is considered a preventive strategy, then this hypothesis can only be supported (1) *if a culture is possible to change*; and (2) *if people are seen as proactive cultural change agents.*

Mainstream theories on cultures may pose a challenge to that assumption. For example, the well-cited framework of Hofstede has dominated many study fields since the 80s (see Kirkman et al., 2006 for a review) and is rooted in the assumption that (1) culture is static, and (2) people are the consequences of their culture. Firstly, culture is static in the sense that national values are “as hard as a country’s geographic position” (Hofstede and Hofstede, 2005, p. 13). These values are unlikely to change across multiple generations, regardless of global movements (see for example critics by Nakata, 2009; McSweeney, 2016). “While change sweeps the surface, the deeper layers remain stable, and the [national] culture rises from its ashes like the phoenix” (Hofstede and Hofstede, 2005, p.36). If changes in values do happen, they occur at a very slow rate, and because *the whole world changes together* at more or less the *same speed*, the gaps between national cultures remain more or less the same (Hofstede, 2001). Based on this assumption, the ranking of countries is valid because cultures tend to change together in unison (but see also Beugelsdijk and Welzel, 2018 for a discussion on cultural change). Secondly, this static paradigm of culture assumes that as a collective, people are the products of their culture. Basic values have been programmed into a person’s mind from young age and remain stable: “We assume that each person carries a certain amount of mental programming which is stable over time” (Hofstede, 1980, p.14). In other words, people are born more or less as blank slates, ready to absorb their first culture in the form of a mental program, hence, Hofstede’s analogy of “culture is the software of the mind.” In sum, the static paradigm of culture posits that as a collective, people are the “consequences” or the product of their culture.

Because Hofstede’s theory is usually employed for teaching cultural awareness and competence, its assumption of “static culture” and “people as a cultural product” may present some obstacles toward the preventive approach that this paper proposes. After all, if values are stable and people are passive, it may seem pointless to advocate for cultivating and aligning with egalitarian values. More broadly, it may seem challenging to advocate for alternative possibilities in which people can proactively exercise human agency in changing their own culture. In other words, viewing people as passive cultural dope may undermine our own role of cultural authority (Swidler, 1986).

In contrast with the static paradigm, there are different cultural frameworks that have been sidelined academically but deserve attention for their interactive approach (Nguyen-Phuong-Mai, 2017b). The dynamic paradigm of culture, for example, posits that culture evolves over time (Adams and Markus, 2001), opposing values coexist (Osland and Bird, 2000), people are active and creative problem solvers who embrace a “strategy of action” (Swidler, 1986) rather than a passive “cultural dope” (Crane, 1994). This dynamic paradigm “restores human agency to social theory” (Forte, 1999, p. 55). Such a proactive approach indicates human’s agency in our relationship with culture. Proposing a shift of paradigm, Nguyen-Phuong-Mai (2019, 2020) argued that humans are both product and producer of culture, in the sense that individuals are influenced by cultures, yet they can also proactively and deliberately be change agents, re-shaping both themselves and the cultures around them. While Hofstede, embracing the static paradigm, insisted that culture is the software of the mind, the dynamic paradigm and its evolving nature suggested that “not culture, but *context* is the software of the mind” (Nguyen-Phuong-Mai, 2017a,^2020^). This notion of a dynamic culture allows the possibility of bias preventive strategies in which people are “rapid niche constructors” (Huebner, 2016). People can build the world so that it reflects what they want in the future. After all, this is also the notion of culture that justifies the old adage: “be the change you want to see.”

## Conclusion

As issues affecting individuals and societies become ever more complex, interdisciplinary research is more needed and valued. This paper conducted a preliminary study of reviewing and exploring potential bias strategies and approaches. It utilized a change management framework as a platform to incorporate insight from studies with successful change interventions in the field of reward management, social neuroscience, health behavioral change, and cognitive behavioral therapy. The broader take-home message is that (1) current bias strategies can be improved and new ones can be developed with insight from diverse study fields that involve change management; (2) a holistic and preventive approach could be sustainable through a multipronged and proactive strategy that targets the collective as a whole; and (3) while people can’t control prejudicial thoughts instantly, they aren’t merely stimulus-response machine, and bias strategies should empower people in the role of change agents who can act proactively to regulate the very environment that gives rise to their own biases.

## Data Availability Statement

The original contributions presented in the study are included in the article/supplementary material, further inquiries can be directed to the corresponding author.

## Author Contributions

The author confirms being the sole contributor of this work and has approved it for publication.

## Conflict of Interest

The author declares that the research was conducted in the absence of any commercial or financial relationships that could be construed as a potential conflict of interest.

## Publisher’s Note

All claims expressed in this article are solely those of the authors and do not necessarily represent those of their affiliated organizations, or those of the publisher, the editors and the reviewers. Any product that may be evaluated in this article, or claim that may be made by its manufacturer, is not guaranteed or endorsed by the publisher.
